# Latent Class Analysis on Types of Challenging Behavior in Persons with Developmental Disabilities: Focusing on Factors Affecting the Types of Challenging Behavior

**DOI:** 10.3390/bs13110879

**Published:** 2023-10-24

**Authors:** Daeyong Kim

**Affiliations:** Department of Behavior Analysis and Intervention, Konyang Cyber University, 158, Gwanjeodong-ro, Seo-gu, Daejeon 35365, Republic of Korea; kimdy1988@naver.com; Tel.: +82-42-722-0095

**Keywords:** activities of daily living, behavioral intervention, challenging behavior, developmental disabilities, latent class analysis

## Abstract

This study sought to analyze the latent classes of challenging behaviors among persons with developmental disabilities and examine the effects of related variables. To this end, the Korea Employment Agency for Persons with Disabilities collected data from the Survey on the Work and Life of Persons with Developmental Disabilities from 3000 households that included at least one family member with a developmental disability aged ≥15 years, surveying the persons themselves as well as their caregivers. As a result of the analysis, four latent classes were derived based on the types of challenging behavior and named as follows: overall challenging behavior, aggressive behavior, socially inappropriate behavior, and no challenging behavior. The main disability, disability grade, presence of multiple disabilities, disability status, activities of daily living, reading skills, writing skills, and situational awareness were significant factors affecting each latent class in the type of challenging behavior. Significant factors differed among the groups. This study identified the types of challenging behaviors and their influencing factors in a large sample of individuals with developmental disabilities and analyzed the correlation between their challenging behaviors and activities of daily living.

## 1. Introduction

Challenging behavior is an umbrella term for many forms of behavior [1] and is defined as socially unacceptable and physically dangerous behavior that negatively affects education, daily life, and social integration [2,3]. However, it is difficult to find a perfectly unified definition of challenging behavior. Challenging behavior is a common difficulty experienced by individuals with developmental disabilities and manifests as non-compliance, self-injury, harming of others, stereotyped behavior, tantrums, crying, and damaging of property; therefore, this behavior covers a wide spectrum from mild, short-lived behavior to severe, chronic, and potentially life-threatening behavior [3]. When diagnosed with developmental disabilities, people exhibit more diverse and seriously challenging behavior than those with psychopathological diseases or atypical development [4]. People with developmental disabilities can encounter major disruptive factors in social integration owing to seriously challenging behavior, including harming others, self-injury, and destructive behavior [5].

Approximately 10–15% of people with developmental disabilities exhibit challenging behavior [6]. According to previous studies, 10–13% of children with developmental disabilities require intensive intervention for challenging behavior [1,7,8]. To formulate service plans for challenging behavior, it is important to identify the objective status, such as the types and frequency of the challenging behavior displayed by individuals with developmental disabilities [9].

Generally, challenging behaviors are classified into the following types: attention deficit and hyperactivity [10], aggressive and antisocial behavior, inappropriate and immature behavior, personality disorder [11], destructive behavior, disruptive behavior, lightly disruptive behavior [12], aggression, destructiveness, self-injurious behavior [13], harming oneself and others, stereotyped behavior, and lack of self-management [14]. People exhibiting challenging behavior are generally known to exhibit a combination of two or more types of challenging behavior [6].

According to the National Survey on Persons with Developmental Disabilities in South Korea, 2021, challenging behavior displayed by persons with developmental disabilities can be classified into six types, with the most common being “self-injurious behavior (30.6%)”, followed by “destroying or taking things away (22.3%)” and “threatening or harassing others (20.9%)”. In particular, persons with autism spectrum disorder (ASD) generally exhibit more challenging behavior than those with intellectual disabilities; however, this research was conducted during the COVID-19 pandemic and should be interpreted with caution [15]. Holden and Gitlesen [16] conducted a study on approximately 900 people with developmental disabilities living in a county in Norway by classifying challenging behavior based on the level of required care. They found that 11.1% of people with developmental disabilities exhibited challenging behavior. More specifically, 6.4% exhibited behaviors that caused harm to others, 2.3% exhibited self-injurious behavior, and 7.1% exhibited destructive or socially unacceptable behavior.

As such, challenging behavior is categorized according to the form of behavior, severity, and level of care required; however, actual interventions for challenging behavior are based on individual characteristics, environmental variables, and behavioral functions that lead to challenging behavior rather than the types of behavior [17,18]. With the increasing awareness that proactive and educational methods are effective in supporting challenging behavior, a more comprehensive preventive approach to positive education is being taken to deal with factors leading to challenging behavior.

Among the factors influencing challenging behavior, individual characteristics such as sex, age, and disability are known to affect challenging behavior [19]. According to previous studies, challenging behavior increases with the severity of intellectual disabilities [20], and people diagnosed with ASD show more frequent challenging behavior than others [16,21]. Moreover, the rate of challenging behavior peaked at 20–40 years of age. This implies that challenging behavior continues into adulthood, and its severity will likely intensify as the person matures physically [16,22].

Positive behavior support (PBS) and applied behavior analysis are the main approaches used to reveal the causes of challenging behavior according to environmental variables and behavioral functions [23]. PBS has garnered considerable endorsements from federal agencies, driving organizational growth, research, and outreach [23]. Its methodologies are widely adopted in human services and educational settings, becoming foundational in state systems and institutions [23]. These two approaches classify the causes of challenging behavior into asking what one needs, self-stimulating behavior for sensory stimulation, avoiding tough situations or tasks, seeking attention, and expressing physical discomfort [24,25]. Hence, challenging behavior is derived from an interaction between the individual characteristics of persons with developmental disabilities and environmental variables. This emphasizes the importance of functional behavioral assessment that identifies environmental causes to intervene in challenging behavior [17,26]. Once the behavioral cause is identified based on the functional behavioral assessment, an intervention is planned and implemented to prevent challenging behavior by changing the setting or antecedent event, intervening with an alternative behavior, or appropriately manipulating the consequence to reduce future occurrences. Among these, setting events are difficult to adjust as they are not temporally close to challenging behavior, such as antecedent events or subsequent outcomes. Therefore, they must be reviewed to prevent challenging behavior. However, it is difficult to elucidate the variables for setting events [12].

In particular, challenging behavior has been reported to be serious if communication or activities of daily living (ADLs) of people with developmental disabilities, which can be regarded as part of the setting event, are limited [1,8]. However, this explanation is limited to fragmentary factors. Moreover, only a few studies have examined the pattern of occurrence of challenging behavior or performed detailed cross-sectional analyses on how factors affect challenging behavior. Recognizing this gap, there is a pressing need to delve deeper into the intricate forms and influencing factors of challenging behaviors in this demographic. Employing a systematic method to understand these behaviors can offer more comprehensive insights. Accordingly, this study classified latent classes based on the forms of challenging behavior displayed by persons with developmental disabilities using latent class analysis, and analyzed the effects of factors that affect classification.

## 2. Materials and Methods

### 2.1. Research Participants

This study used data from the Survey on the Work and Life of Persons with Developmental Disabilities, provided by the Korea Employment Agency for Persons with Disabilities. The Survey on the Work and Life of Persons with Developmental Disabilities is a cross-sectional survey that examines factors affecting the work and life of persons with developmental disabilities and their caregivers. The survey included 3000 households in South Korea as of 15 May 2021, consisting of individuals with developmental disabilities and their caregivers (*n* = 3000). Each household included at least one family member aged ≥ 15 years with a developmental disability, with an average age of 32.7 (standard deviation, 14.3) years. Of them, 2044 were male, and 956 were female. Pertaining to the specific disabilities, 1998 individuals had intellectual disabilities, whereas 1002 had ASD. Individuals with developmental disabilities are legally registered with intellectual disabilities and ASD according to the Act on the Welfare of Persons with Disabilities. For the population and sampling frame, a list of registered persons with disabilities as of December 31 of the previous year was used. This study used the data of 1998 persons with intellectual disabilities and 1002 persons with ASD with their caregivers’ responses from the 2021 Survey on the Work and Life of Persons with Developmental Disabilities data and questionnaire released on 31 March 2022. This study was approved by the Institutional Review Board of Konyang University (approval number: KYU 2023-03-047). Given its retrospective nature and anonymized data, the requirement for informed consent was waived. Our team strictly adhered to research ethics, emphasizing the protection of participants’ personal information and rights.

### 2.2. Measurement Tool

Variables related to challenging behavior included self-injurious behavior, behavior causing harm to others, destructive behavior, disruptive behavior, abnormal repetitive behavior, socially aggressive behavior, withdrawn or inattentive behavior, and uncooperative behavior, with the responses including 1 = Never, 2 = Sometimes, and 3 = Often. To focus on examining the types of challenging behavior without considering the frequency, “Never” was dummy coded with 0 = No, while “Sometimes” and “Often” were dummy coded with 1 = Yes. Individual characteristics, ADLs, communication, literacy, and situational awareness were included as factors affecting the classification. Sex was dummy coded as 0 = female and 1 = male. The main disability was dummy coded as 0 = intellectual disability and 1 = ASD. The disability grade was coded as 0 = Grade 1, 1 = Grade 2, and 2 = Grade 3; the presence of multiple disabilities was dummy coded as 0 = without multiple disabilities and 1 = with multiple disabilities; and the disability status was coded as 1 = gradually improving, 2 = not improving or deteriorating, and 3 = gradually deteriorating. ADLs included 16 items; 1 item was deleted from the exploratory factor analysis, after which 3 factors were presented. Details of the variables used in the analysis are listed in Table 1.

The first factor consisted of items related to changing clothes, washing face/brushing teeth/washing hair, taking a bath, eating when served, walking, defecating and urinating, and were rated on a 4-point Likert scale from “Totally dependent” to “Independent”. The mean of the six items was calculated, and the reliability (Cronbach’s alpha) was 0.951. The second factor consisted of three items: cleaning, preparing meals, and laundry. The mean of these items was calculated and the reliability of the items (Cronbach’s alpha) was 0.940. The third factor consisted of six items: going out nearby, using public transportation, purchasing things, managing money, using a phone, and taking medication. The mean of these items was calculated and the reliability of the items (Cronbach’s alpha) was 0.957. For communication, understanding what others say was rated on a 4-point Likert scale and coded as follows: 1 = can understand two or more sentences; 2 = can understand a simple sentence; 3 = can understand words only; and 4 = can barely understand others. Expressing opinions verbally was rated on a 5-point Likert scale and coded as follows: 1 = expressing opinions in at least two words or in sentences; 2 = expressing opinions using clear words; 3 = expressing opinions using unclear words; 4 = expressing opinions using unclear sounds; and 5 = cannot express any opinion with sounds at all. Understanding and using nonverbal expressions were rated on a 3-point Likert scale. The reliability (Cronbach’s alpha) of all communication items was 0.881. Literacy, which includes reading and writing, was coded with 1 = Impossible, 2 = Possible on a limited basis, and 3 = Possible. The reliability of the items (Cronbach’s alpha) was 0.970. Situational awareness consisted of three items related to awareness of location and place, awareness of people around them, and awareness of the situation, and was rated on a 4-point Likert scale. The reliability of the items (Cronbach’s alpha) was 0.938.

### 2.3. Data Analysis

**Figure 1:**
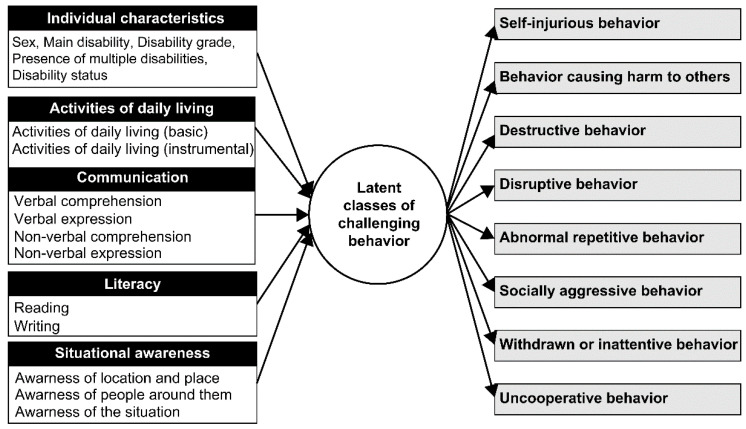
Research model. Figure 1 depicts the research model, utilizing types of challenging behaviors as variables to explore latent classes. Additionally, individual characteristics, activities of daily living, communication skills, literacy, and situational awareness are considered as influencing factors.

This study used types of challenging behavior (self-injurious behavior, behavior causing harm to others, destructive behavior, disruptive behavior, abnormal repetitive behavior, socially aggressive behavior, withdrawn or inattentive behavior, and uncooperative behavior) as variables to explore latent classes according to the type of challenging behavior, and used individual characteristics (sex, main disability, disability grade, presence of multiple disabilities, and disability status), ADLs, communication skills, literacy, and situational awareness as influencing factors. Figure 1 shows the details of the research model.

This study conducted a latent class analysis and classified the types of challenging behaviors in individuals with developmental disabilities using Mplus 8.3. When determining the number of latent profiles, models were compared based on information indices, model comparison validation, and classification quality while increasing the number of groups (two to six groups). The final model that most closely matched the data was selected. First, entropy, which represents the quality of classification, was used. Generally, the model was considered a fit when the entropy was ≥0.8 [27]. Next, information indices, such as the Akaike information criterion (AIC), Bayesian information criterion (BIC), and sample-size adjusted BIC, were used to compare the model fit. Lower information indices indicated a better model fit [28,29,30]. Finally, the Lo–Mendell–Rubin adjusted likelihood ratio test and the parametric bootstrap likelihood ratio test were used [31,32]. If the *p*-value in the test was significant, k latent class models were adopted. If not significant, (k − 1) latent class models were selected. After determining the number of latent classes, the three-step approach proposed by Asparouhov and Muthén [33] was used to verify the influencing factors.

Multinomial logistic analysis was conducted to identify the factors affecting the classification of each latent class and to derive the influence of each factor. One class was set as the reference group and was compared with the other classes. This study examined the variables that were significant factors of each latent class among individual characteristics, ADLs, communication skills, literacy, and situational awareness.

## 3. Results

### 3.1. Latent Class Analysis According to the Types of Challenging Behavior

A latent class analysis was conducted to identify the characteristics of each class. The number of latent classes increased a level from two to determine the number of latent classes while examining information indices, validation of model comparison, classification rate, and entropy that represent the quality of classification. The results are presented in Table 2.

**Figure 2:**
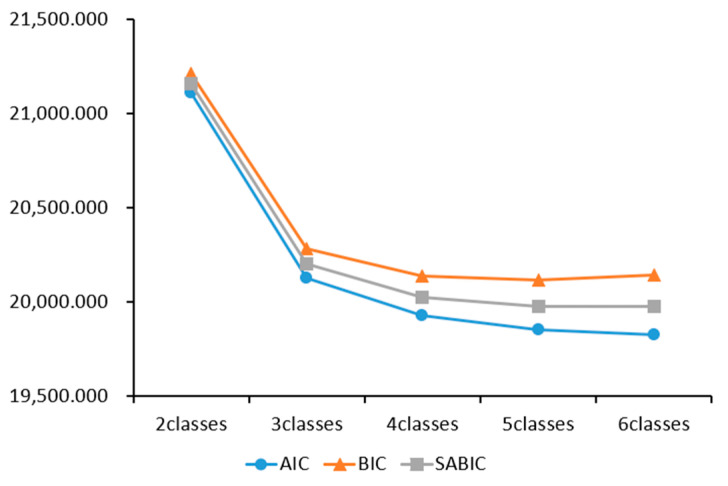
Elbow plots for the Akaike information criterion (AIC), Bayesian information criterion (BIC), and sample-size adjusted Bayesian information criterion (SABIC). Figure 2 displays the model fit results. While information indices (AIC, BIC, and sample-size adjusted BIC) generally decreased with increasing latent classes, a visual inflection in the graph suggested the 4-class model as the optimal one.

First, the model with two to four latent classes showed a good fit with an entropy > 0.800. Second, the AIC, BIC, and sample-size adjusted BIC, which are information indices, tended to decrease as the number of latent classes increased. In this case, the number of latent classes was indicated where the visual graph of the information indices began to show a gentle curve. Since the values of all information indices gradually decreased when the number of latent classes increased from four to five, the model with four latent classes has the best fit, and the results are as shown in Figure 2. Third, the validation of the model comparison in the Lo–Mendell–Rubin adjusted likelihood ratio test and the bootstrap likelihood ratio test was significant when there were two to five latent classes. Considering the classification criteria presented in Table 2, the interpretability of latent classes, and the model simplicity derived from each model, it seems most suitable to classify the types of challenging behavior in persons with developmental disabilities into four classes.

Second, the quality of the latent class classification was inspected using the posterior probabilities of the classes. The classification was considered accurate when the posterior probability was ≥0.700 [34]. The values of the average posterior probability shown in the table indicate that the classification was accurate for Class 1, 93.3%; Class 2, 77.2%; Class 3, 83.8%; and Class 4, 94.6%; the results are as shown in Table 3.

**Figure 3:**
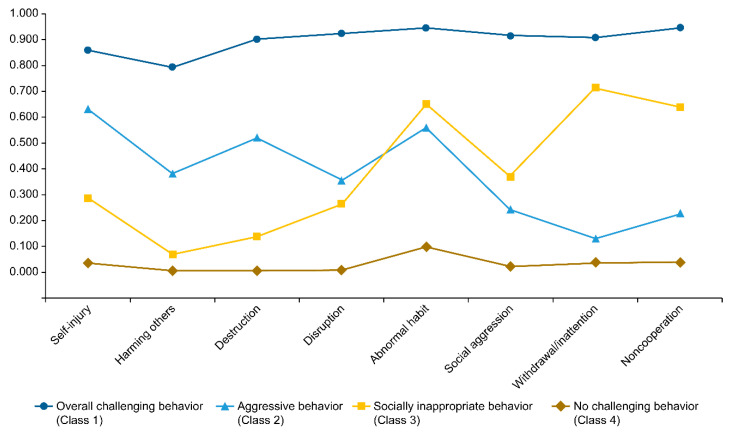
Latent classes of challenging behaviors. “Overall Challenging Behavior” (16.4% of individuals) displays a range of challenging behaviors. “Aggressive Behavior” (11.0% of individuals) predominantly shows self-injurious, harmful, and destructive behaviors. “Socially Inappropriate Behavior” (20.9% of individuals) includes lesser aggressive behaviors than Class 2 but more abnormal repetitive and socially aggressive behaviors. “No Challenging Behavior” (51.7% of individuals) displays minimal indicators of challenging behaviors. Both Classes 2 and 3 exhibited abnormal repetitive behaviors.

Third, the characteristics of the optimal model with four latent classes were estimated. The hierarchical results according to the types of challenging behavior derived in the final model for the analysis of each latent class are shown in Figure 3. The coefficients and standard errors for each type of challenging behavior are shown in Table 4.

The latent class patterns based on the types of challenging behaviors are shown in Figure 3. Considering the coefficients, the groups were named overall challenging behavior, aggressive behavior, socially inappropriate behavior, and no challenging behavior. For the characteristics of each group, approximately 16.4% of persons with developmental disabilities belonged to Class 1, showing all kinds of challenging behavior, such as self-injurious behavior, behavior causing harm to others, destructive behavior, socially aggressive behavior, and uncooperative behavior, thereby named as “overall challenging behavior”. Approximately 11.0% of persons with developmental disabilities belonged to Class 2, showing high levels of self-injurious behavior, behavior causing harm to others, and destructive behavior compared to other challenging behaviors. Therefore, they were considered as having aggressive behavior. Approximately 20.9% of individuals with developmental disabilities belonged to Class 3, showing less aggressive behavior than Class 2 but more abnormal repetitive behavior, socially aggressive behavior, withdrawn or inattentive behavior, and uncooperative behavior. Therefore, they were considered as having socially inappropriate behavior. Approximately 51.7% of individuals with developmental disabilities belonged to Class 4, showing low coefficients for all challenging behaviors. Thus, they were considered as having no challenging behavior. Both Classes 2 and 3 exhibited abnormal repetitive behaviors.

### 3.2. Analysis of Factors Affecting Classification

The results of multinomial logistic analysis for identifying the factors affecting the classification of each latent class and for deriving each factor’s influence are shown in Table 5.

First, when the no challenging behavior class was set as the reference group, the main disability, disability grade, presence of multiple disabilities, disability status, ADLs, literacy (reading and writing), and situational awareness of individual characteristic variables were significant when compared with other classes.

When focusing on individual characteristics, people with ASD were more likely to belong to the socially inappropriate behavior, aggressive behavior, and overall challenging behavior groups than to the no challenging behavior reference group.

Moreover, people with higher disability grades were more likely to belong to the aggressive behavior and overall challenging behavior groups than to the no challenging behavior reference group. Those with multiple disabilities and more severe disability status were more likely to belong to the overall challenging behavior group.

For ADLs, people who need more help from others in ADLs, such as changing clothes, washing their face/brushing teeth/washing hair, taking a bath, eating when served, walking, and defecating and urinating, were more likely to belong to the socially inappropriate behavior group than to the no challenging behavior reference group. Those who needed more help in ADLs such as going out to somewhere nearby, using public transportation, purchasing things, managing money, using a phone, and taking medication, were more likely to belong to the socially inappropriate behavior and overall challenging behavior groups.

Communication skills, such as verbal comprehension, verbal expression, nonverbal comprehension, and nonverbal expression, did not show significant results, with no challenging behavior as the reference group. Regarding reading skills, people who could read Korean were more likely to belong to the socially inappropriate behavior, aggressive behavior, and overall challenging behavior groups than the no challenging behavior group. Regarding writing skills, those who could not write Korean were more likely to belong to the overall challenging behavior group than to the no challenging behavior group.

Finally, people with lower means of awareness of location and place, awareness of people around them, and awareness of the situation were more likely to belong to the socially inappropriate behavior and overall challenging behavior groups than to the no challenging behavior group.

When the socially inappropriate behavior class was set as the reference group, individual characteristic variables, such as main disability, disability grade, disability status, and ADLs, showed significance in the comparison between classes. Those whose main disability was ASD had a higher disability grade, and whose disability status was deteriorating were more likely to belong to the overall challenging behavior group than to the socially inappropriate behavior group. Those who needed more help with ADLs, such as changing clothes, washing face/brushing teeth/washing hair, taking a bath, eating when served, walking, defecating, and urinating, were more likely to belong to the aggressive behavior group and the overall challenging behavior group compared to the socially inappropriate behavior group.

When the aggressive behavior class was set as the reference group, individual characteristic variables, such as disability status and having multiple disabilities, were identified as significant influencing factors. People with multiple disabilities and whose disability status deteriorated were more likely to belong to the overall challenging behavior group than to the aggressive behavior group.

## 4. Discussion

This study classified the types of challenging behavior displayed by persons with developmental disabilities into latent subgroups using data from the Survey on the Work and Life of Persons with Developmental Disabilities provided by Korea Employment Agency for Persons with Disabilities. Differences in group characteristics were examined after analyzing the effects of the variables on each class.

First, four latent classes were most suitable according to the latent class analysis on the types of challenging behavior, including “overall challenging behavior (16.4%)”, “aggressive behavior (11.0%)”, “socially inappropriate behavior (20.9%)”, and “no challenging behavior (51.7%)”. The overall challenging behavior group showed a high occurrence of all eight types of challenging behaviors examined in the Survey on the Work and Life of Persons with Developmental Disabilities. The aggressive behavior group showed challenging behaviors such as self-injurious behavior, behavior causing harm to others, destructive behavior, and disruptive behavior. The socially inappropriate behavior group showed challenging behaviors such as abnormal repetitive behavior, socially aggressive behavior, withdrawn or inattentive behavior, and uncooperative behavior. Among the types of challenging behaviors, disruptive behavior was high in both the aggressive and socially inappropriate behavior groups.

Second, individual characteristics, such as the main disability, disability grade, presence of multiple disabilities, and disability status, were identified as affecting the occurrence of challenging behaviors. By setting the no challenging behavior group as the reference group and using the overall challenging behavior, aggressive behavior, socially inappropriate behavior, and no challenging behavior groups as comparison groups for analysis, it was found that people with ASD tended to show more severe challenging behavior than those with intellectual disabilities, which is consistent with the results of McClintock et al. [21] and Myrbakk and Tetzchner [35]. This also supports the findings of previous studies reporting that people with ASD show challenging behavior [36].

In particular, this study showed that people with higher disability grades were more likely to belong to the aggressive and overall challenging behavior groups. Disability grading was abolished in South Korea; however, before 2019, people with Grade 1 ASD had an IQ score of 70 or below and a Goal Attainment Scale (GAS) score of 20 or below, whereas those with Grade 2 ASD had an IQ of 70 or below and a GAS score between 21 and 40. This is consistent with the report by Holden and Gitlesen [16], who argued that people with more severe ASD and intellectual disabilities also showed more challenging behaviors. Moreover, this study proved that people with multiple disabilities are more likely to belong to the overall challenging behavior group. The multiple disabilities examined in the Survey on the Work and Life of Persons with Developmental Disabilities include brain lesions, physical disabilities, language disorders, and mental disorders. People with such disabilities are more likely to show overall challenging behaviors. However, as it was difficult to confirm whether ASD was accompanied by disabilities, such as depression, anxiety, and attention deficit hyperactivity disorder, these results should be interpreted carefully.

Third, the aggressive and socially inappropriate behavior groups showed a high level of abnormal habits among challenging behaviors. Considering that repetitive and stereotyped behaviors are core features of ASD, it seems natural that abnormal habits, such as repetitive and stereotyped behaviors, appear frequently [37]. Considering the results of this study, the assumption that people diagnosed with ASD are more likely to belong to a group with challenging behaviors seems reasonable.

Fourth, communication skills were not highly significant for challenging behaviors. This result is contrary to the argument that the absence of certain skills, such as a delay in social and communication skills, may increase challenging behavior [21,38], and that persons with developmental disabilities whose communication skills and social development are limited display more serious challenging behavior [1,8]. In particular, factors related to overall communication, such as verbal comprehension and expression or nonverbal comprehension and expression, did not show significant effects on the possibility of belonging to classes showing challenging behavior; however, people with literate reading skills were more likely to belong to the group showing challenging behavior, and those with no writing skills were significantly more likely to belong to the group showing overall challenging behavior.

Fifth, people are more likely to belong to groups that show challenging behaviors depending on their level of ADLs. Moreover, even if they belonged to the overall challenging behavior or aggressive behavior groups, those with improved ADLs were more likely to belong to the group with a lower severity of challenging behavior. This is consistent with the results of previous studies, in which people with developmental disabilities who showed aggressive behavior had lower social skills [39,40].

These results lay the grounds for viewing the challenging behaviors displayed by persons with developmental disabilities as a means of performing ADLs. Abilities related to daily life can be divided into basic ADLs and instrumental ADLs. Basic ADLs are related to survival, including changing clothes, washing/brushing teeth, washing hair, taking a bath, eating when served, walking, defecating, and urinating. Instrumental ADLs are necessary for home and social life, including going out, using public transportation, purchasing items, managing money, using a phone, and taking medication [41,42,43]. Generally, when intervening for individuals with challenging behaviors, one of the most effective intervention methods is to use communication skills. The results of this study confirm that teaching ADLs significantly prevents challenging behaviors. Evidence-based practice, known as functional communication training, is a way to satisfy the needs, demands, and interests expressed through challenging behaviors by expressing them in a socially acceptable way, such as spoken language. If the intention to perform daily life activities is conveyed in challenging behaviors, efforts must be made to analyze and teach ADLs to set the direction for behavioral intervention.

However, this study had limitations in that the data were based on reports from caregivers. Because of the cognitive nature of persons with developmental disabilities, proxy responses from caregivers are inevitable. However, even if caregivers are considered to know people with developmental disabilities very well, discrepancies with reality are inevitable in subjective domains that are not superficial [44]. Therefore, a careful interpretation of the study results is necessary. Myrbakk and von Tetzchner [35] reported that a survey of challenging behaviors targeting people with developmental disabilities effectively compared the effectiveness of each service conducted in different countries or regions within the same country. Based on this, the results of the current study can serve as the foundation for a detailed analysis of services related to challenging behaviors. In future research, exploring other factors influencing challenging behaviors that were not addressed in this study would be beneficial. Moreover, delving into the discrepancies between caregiver perceptions and the actual experiences of individuals with developmental disabilities might offer a more comprehensive understanding of the topic. In this study, legal standards were used to classify the main disability. Accordingly, individuals with both severe autism and intellectual disability were diagnosed as having autism spectrum disorder, and an additional disability grade ranging from 1 to 3 was assigned to indicate the coexistence. This method has the advantage of allowing for a clear classification based on specific criteria; however, it is limited in fully reflecting the diverse symptoms and characteristics of each individual. Therefore, further research and discussions on this classification method will be needed in the future.

## Figures and Tables

**Table 1:** Variables used in the analysis.

Classification		Variable Name	Variable Content
Type of challenging behavior		Self-injurious behavior (self-injury)	0 = No, 1 = Yes
		Behavior causing harm to others (harming others)	
		Destructive behavior (destruction)	
		Disruptive behavior (disruption)	
		Abnormal repetitive behavior (abnormal habit)	
		Socially aggressive behavior (social aggression)	
		Withdrawn or inattentive behavior (withdrawal/inattention)	
		Uncooperative behavior (noncooperation)	
Determinant	Individual characteristics	Sex	0 = Female, 1 = Male
		Main disability	0 = intellectual disabilities, 1 = ASD
		Disability grade	0 = Grade 1, 1 = Grade 2, 2 = Grade 3
		Presence of multiple disabilities	0 = Without multiple disabilities 1 = With multiple disabilities
		Disability status	1 = Gradually improving 2 = Not improving or deteriorating 3 = Gradually deteriorating
	Activities of daily living	Changing clothes, washing face/brushing teeth/washing hair, taking a bath, eating when served, walking, defecating and urinating	The mean of six items on activities of daily living 1 = Totally dependent 2 = Substantially dependent 3 = Partially dependent 4 = Independent
		Cleaning, preparing meals, doing the laundry	The mean of three items on activities of daily living 1 = Totally dependent 2 = Substantially dependent 3 = Partially dependent 4 = Independent
		Going out to somewhere nearby, using public transportation, purchasing things, managing money, using a phone, taking medication	The mean of six items on activities of daily living 1 = Totally dependent 2 = Substantially dependent 3 = Partially dependent 4 = Independent
	Communication	Understanding what others say	1 = Can understand two or more sentences 2 = Can understand a simple sentence 3 = Can understand words only 4 = Can barely understand others
		Expressing opinions verbally	1 = Expressing opinions in at least two words or in sentences 2 = Expressing opinions using clear words 3 = Expressing opinions using unclear words 4 = Expressing opinions making unclear sounds 5 = Cannot express any opinion with sounds at all
		Understanding nonverbal expressions	1 = Can understand 2 = Can understand on a limited basis 3 = Cannot understand
		Using nonverbal expressions	1 = Can use 2 = Can use on a limited basis 3 = Cannot use
	Literacy	Reading	1 = Impossible, 2 = Possible on a limited basis, 3 = Possible
		Writing	1 = Impossible, 2 = Possible on a limited basis, 3 = Possible
	Situational awareness	Awareness of location and place Awareness of people around them Awareness of the situation	The mean of three items concerning cognitive ability 1 = Totally dependent 2 = Substantially dependent 3 = Partially dependent 4 = Independent

**Table 2:** Model fit indices of the latent class analysis.

		Two Classes	Three Classes	Four Classes	Five Classes	Six Classes
Information index	AIC	21,110.815	20,128.954	19,927.179	19,850.558	19,825.809
	BIC	21,212.867	20,285.033	20,137.285	20,114.692	20,143.970
	SABIC	21,158.851	20,202.421	20,026.077	19,974.887	19,975.568
Quality of classification	Entropy	0.895	0.830	0.806	0.758	0.755
Validation of model comparison	LMRLRT	0.000	0.000	0.000	0.000	0.039
	BLRT	0.000	0.000	0.000	0.000	0.000
Classification rate	Class 1	0.311	0.330	0.164	0.170	0.123
	Class 2	0.069	0.510	0.109	0.126	0.027
	Class 3		0.160	0.517	0.133	0.437
	Class 4			0.209	0.115	0.124
	Class 5				0.456	0.139
	Class 6					0.150

AIC, Akaike information criterion; BIC, Bayesian information criterion; BLRT, bootstrap likelihood ratio test; LMRLRT, Lo–Mendell–Rubin adjusted likelihood ratio test; SABIC, sample-size adjusted Bayesian information criterion.

**Table 3:** Most likely, the latent class membership (row) by latent class (column).

	Class 1	Class 2	Class 3	Class 4
Probability that the behavior would belong to Class 1	0.933	0.023	0.044	0.000
Probability that the behavior would belong to Class 2	0.038	0.772	0.147	0.043
Probability that the behavior would belong to Class 3	0.024	0.089	0.838	0.048
Probability that the behavior would belong to Class 4	0.000	0.022	0.032	0.946

**Table 4:** The coefficients and standard errors for each type of challenging behavior.

Variable	Class 1		Class 2		Class 3		Class 4	
	Overall Challenging Behavior		Aggressive Behavior		Socially Inappropriate Behavior		None	
	Coefficient	Standard Error	Coefficient	Standard Error	Coefficient	Standard Error	Coefficient	Standard Error
Self-injury	0.858	0.020	0.631	0.049	0.286	0.039	0.036	0.007
Harming others	0.791	0.026	0.381	0.050	0.068	0.021	0.006	0.003
Destruction	0.899	0.021	0.519	0.063	0.139	0.031	0.006	0.004
Disruption	0.922	0.017	0.355	0.051	0.263	0.031	0.007	0.003
Abnormal habit	0.945	0.014	0.560	0.049	0.649	0.029	0.098	0.010
Social aggression	0.914	0.018	0.241	0.043	0.369	0.028	0.021	0.005
Withdrawal/inattention	0.907	0.019	0.129	0.077	0.713	0.038	0.036	0.008
Noncooperation	0.945	0.015	0.225	0.066	0.637	0.035	0.040	0.007
Percentage	16.4%		11.0%		20.9%		51.7%	

**Table 5:** Multinomial logistic regression results.

**Reference Group**	**No Challenging Behavior**					
**Comparative Group**	**Socially Inappropriate Behavior**		**Aggressive Behavior**		**Overall Challenging Behavior**	
**Variable**	**Coefficient**	**Standard Error**	**Coefficient**	**Standard Error**	**Coefficient**	**Standard Error**
Sex	0.021	0.140	−0.131	0.222	0.230	0.154
Main disability	0.882 ***	0.150	1.351 ***	0.206	1.713 ***	0.154
Disability grade	−0.103	0.110	−0.504 **	0.171	−0.462 ***	0.111
Multiple disabilities	−0.042	0.219	−0.353	0.364	0.417 *	0.200
Disability status	0.164	0.116	−0.152	0.162	0.519 ***	0.119
Activities of daily living 1	0.464 **	0.145	−0.099	0.169	0.103	0.107
Activities of daily living 2−1	−0.517 ***	0.095	−0.259	0.170	−0.541 ***	0.098
Activities of daily living 2−2	−0.047	0.115	−0.082	0.200	−0.153	0.115
Verbal comprehension	0.088	0.131	−0.135	0.171	0.087	0.121
Verbal expression	−0.026	0.101	−0.036	0.141	0.181	0.095
Nonverbal comprehension	0.175	0.173	0.269	0.275	−0.198	0.167
Nonverbal expression	−0.301	0.164	−0.355	0.242	−0.181	0.164
Reading	0.633 **	0.219	1.090 **	0.340	0.891 ***	0.217
Writing	−0.276	0.221	−0.381	0.354	−0.558 *	0.218
Situational awareness	−0.609 ***	0.152	−0.341	0.233	−0.549 **	0.160
**Reference Group**	**Socially Inappropriate Behavior**					**Aggressive Behavior**
**Comparative Group**	**Aggressive Behavior**		**Overall Challenging Behavior**		**Overall Challenging Behavior**	
**Variable**	**Coefficient**	**Standard Error**	**Coefficient**	**Standard Error**	**Coefficient**	**Standard Error**
Sex	−0.153	0.262	0.209	0.181	0.362	0.243
Main disability	0.470	0.242	0.832 ***	0.175	0.362	0.227
Disability grade	−0.401	0.208	−0.358 **	0.135	0.042	0.188
Multiple disabilities	−0.311	0.420	0.459	0.246	0.770 *	0.365
Disability status	−0.316	0.194	0.354 **	0.136	0.670 ***	0.177
Activities of daily living 1	−0.562 *	0.222	−0.360 **	0.136	0.202	0.168
Activities of daily living 2-1	0.259	0.197	−0.023	0.112	−0.282	0.181
Activities of daily living 2-2	−0.035	0.233	−0.106	0.135	−0.071	0.205
Verbal comprehension	−0.223	0.203	−0.001	0.140	0.221	0.177
Verbal expression	−0.010	0.162	0.207	0.108	0.217	0.141
Nonverbal comprehension	0.094	0.334	−0.373	0.205	−0.467	0.285
Nonverbal expression	−0.054	0.297	0.120	0.202	0.174	0.260
Reading	0.457	0.403	0.259	0.252	−0.199	0.360
Writing	−0.104	0.425	−0.281	0.256	−0.177	0.380
Situational awareness	0.268	0.275	0.060	0.190	−0.208	0.247

* *p* < 0.05, ** *p* < 0.01, *** *p* < 0.001.

## Data Availability

The datasets used and/or analyzed during the current study are available from the corresponding author on reasonable request.
